# Autophagy and unfolded protein response (UPR) regulate mammary gland involution by restraining apoptosis-driven irreversible changes

**DOI:** 10.1038/s41420-018-0105-y

**Published:** 2018-10-15

**Authors:** Anni Wärri, Katherine L. Cook, Rong Hu, Lu Jin, Alan Zwart, David R. Soto-Pantoja, Jie Liu, Toren Finkel, Robert Clarke

**Affiliations:** 1 Department of Oncology and Lombardi Comprehensive Cancer Center, Georgetown University Medical Center, Washington, DC 20057 USA; 2 Institute of Biomedicine, University of Turku Medical Faculty, Turku, 20014 Finland; 3 Department of Surgery and Comprehensive Cancer Center, Wake Forest University, Winston Salem, NC 27157 USA; 4 National Heart Lung and Blood Institute, National Institutes of Health, Bethesda, MD 20892 USA; 5 University of Pittsburgh, UPMC Aging Institute, Pittsburgh, PA 15213 USA

**Keywords:** Preclinical research, Experimental models of disease, Reprogramming, Molecular biology, Preclinical research

## Abstract

The postnatal mammary gland undergoes repeated cycles of proliferation and cell death, most notably when the fully differentiated (lactating) gland dedifferentiates to a prelactation state. Accumulation of milk proteins in the secretory epithelium creates the stress signal that triggers this process (involution). How this stress is perceived, and the cellular processes that are subsequently activated, remain unclear. We now report that Unfolded Protein Response (UPR), autophagy, and apoptosis related genes cluster separately during lactation and involution in the mouse mammary gland. Time-course experiments in rodents show that autophagy and UPR signaling are tightly co-regulated at the transition from reversible to irreversible involution. Inhibition of autophagy by chloroquine or genetic deletion of one ATG7 allele enhanced progression of mammary involution into the irreversible phase, as characterized by an early/precocious induction of apoptosis. These are the first preclinical in vivo data in support of a clinical trial testing an autophagy inhibitor for prevention of intraductal breast malignancy progression to invasive breast cancer. In marked contrast, stimulation of autophagy by low dose tunicamycin treatment reduced apoptosis and extended the reversible phase of involution by sustaining the secretory epithelium. Autophagy stimulators could be used short-term to promote lactation in women experiencing difficulties or irregularities in nursing. Taken together, these data indicate that UPR and autophagy play a key role in regulating the balance between cell survival and apoptosis during normal mammary gland regression.

## Introduction

The mammary gland is a unique tissue in its ability to undergo repeated cycles of cell proliferation, differentiation, death, and tissue remodeling during and after puberty, and during the process of involution that occurs after cessation of lactation. Involution is a normal process by which the terminally differentiated gland transitions, through de-differentiation, to a quiescent state similar to the prepregnancy gland^1,2^. This process is of particular interest, since mammary involution has been implicated as a key contributor to pregnancy-associated breast cancer^3,4^, and a higher metastatic potential^5^. Involution can be divided into two distinct phases: the first phase, 0–48 h (in mouse), is initiated by local factors triggered by milk accumulation in the gland due to cessation of suckling^6,7^. Cell and tissue architecture are maintained, involution is reversible, and lactation can resume with suckling. The second phase (48–144 h in mouse) is regulated by systemic hormones and is irreversible; tissue architecture is destroyed and a robust remodeling is accompanied by adipogenesis^8^. Apoptosis occurs during both phases, triggered by detachment of luminal epithelial cells from the basement membrane^9^.

Autophagy has gained increasing attention in cancer research, including breast cancer, as a key mechanism cancer cells use to cope with cellular stress and produce energy^10^. In contrast, in normal mammary gland physiology, the role of autophagy as a cell survival or cell death process has remained controversial^11,12^. Autophagy has been implicated in the formation of growing ducts during early mammary gland development, and in the mature gland during the formation of epithelial acini in both the bovine^13^ and murine mammary glands^9^. In *Beclin (Becn)1* null mouse embryos, widespread cell death occurs and is embryonic lethal^14^, suggesting a prosurvival role for autophagy during early development. *Becn1*^+/−^ heterozygote mice develop spontaneous tumors and mammary hyperplasias, consistent with a haploinsufficient tumor suppressor role^14^. In contrast, increased *Becn1* expression has been described at the end of lactation in the adult mammary gland and interpreted to imply a cellular prodeath role^12^. During involution, autophagy may exhibit either a prosurvival^9,15^ or prodeath function^16^.

A complex interaction exists among autophagy, the unfolded protein response (UPR), and cell fate outcomes in breast cancer^17,18^. UPR, which is induced by the accumulation of unfolded/misfolded proteins in the endoplasmic reticulum (EnR), can regulate autophagy. Accumulation of milk proteins in the secretory mammary epithelium may create the stress signal that triggers involution, perhaps through the integration of UPR signaling, autophagy, and apoptosis in mammary gland involution. An increase in unfolded/misfolded proteins in the EnR lumen activates the UPR by causing the dissociation of glucose regulated protein 78 (GRP78) from the three UPR signaling arms: PKR-like endoplasmic reticulum kinase (PERK), activating transcription factor 6 (ATF6), and inositol-requiring enzyme 1 (IRE1)^19^. Once released from GRP78, PERK dimerizes and autophosphorylates to become active, resulting in phosphorylation of eIF2α and a halt in cap-dependent protein translation, favoring ATF4 synthesis. Activated ATF4 regulates the transcription of several genes including GRP78, autophagy-related gene 12 (ATG12), and the proapoptotic protein DNA-damage-inducible transcript 3 (DDIT3, also known as GADD153 or CHOP). ATF6 translocates from the EnR to the Golgi complex where site 1 and site 2 proteases cleave ATF6 to produce its transcriptionally active form (cleaved ATF6) that promotes transcription of X-box binding protein 1 (XBP1) and GRP78. When activated by release from GRP78, IRE1 dimerizes, autophosphorylates, and its unique endonuclease becomes active and, among other functions, removes a 26 nucleotide base pair fragment from XBP1. This unconventional splicing action forms trancriptionally active XBP1-Spliced (XBP1-S). XBP1-S then promotes the transcription of p58^IPK^ (negative feedback loop to inhibit PERK signaling), lipid biogenesis proteins, and EnR-associated protein degradation components. The UPR signaling cascade can also occur first with PERK activation, followed by ATF6 cleavage, and lastly XBP1-S formation. In the lactating mammary gland, UPR pathways PERK-eIF2α-ATF4-CHOP^15^ and XBP1^20^ have been implicated in prosurvival and lipogenic functions, respectively.

In this study, we used published gene expression microarray datasets from different phases of mouse mammary gland development, including lactating and involuting samples, and analyzed the expression of apoptosis, autophagy, and UPR genes. We obtained involution time-course samples from wild-type mice using forced weaning. Mice also were treated with and without drug interventions: autophagy stimulating (tunicamycin), inhibiting (chloroquine) and control treatments. We also used a genetic mouse model of ATG7 haploinsufficiency (Atg7^+/−^ vs. Atg7^+/+^). We show here, to our knowledge for the first time, that UPR and autophagy are essential for survival of the terminally differentiated mammary epithelium in the initial (reversible) phase of involution by restraining apoptosis-driven irreversible changes and regression of the epithelium.

## Results

### PCA revealed separation of different time points in involution process

**Fig. 1:**
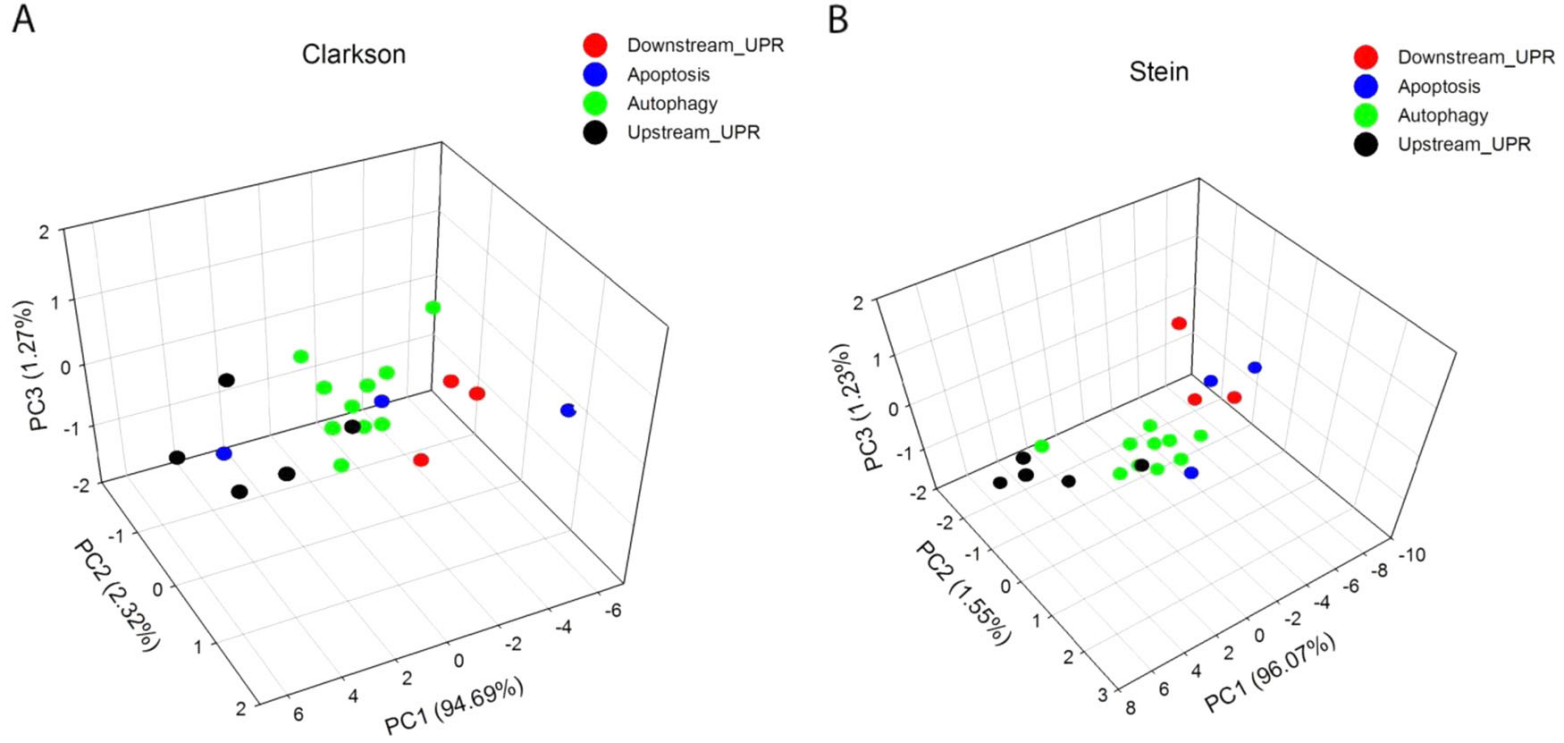
Principal component analysis (PCA) of gene expression microarray data sets. Two published gene expression array data sets (A, B^21, 22^) were used. Data from these studies were derived from nonpooled samples. Multiple time-points of mouse mammary gland developmental stages, including lactation and involution, were analyzed. Datasets were acquired and analyzed as explained in Materials and methods. PCA analysis was performed on involution samples for a 21 gene profile representing apoptosis, autophagy, and UPR (Table 1; Supplementary Table S2) on each data set (**a**, **b**). A three-dimensional biplot shows the separation of apoptosis, autophagy, and UPR/downstream of UPR genes expressed at the different time points during mammary gland involution. Additional visual angles to show clustering of apoptosis genes better are presented in Supplementary Figure S2

To study the expression of UPR, autophagy, and apoptosis genes in the mammary gland, first we searched published gene expression microarray datasets obtained at different stages of (mouse) mammary development; only studies containing involution with several data points (no pooled samples) were selected^21,22^ and analyzed as presented in Materials and methods. We assigned these genes to different groups based on their pathway membership as annotated in Gene Ontology (GO; Autophagy Gene Ontology GO:0006914, Apoptosis Process GO:0006915 and Response to unfolded protein GO:0006986; Table 1; Supplementary Table S2). Principal component analysis (PCA) shows that UPR (internal), UPR (downstream), autophagy, and apoptosis genes, especially the first three, clustered within their own functional group in both datasets during lactation-involution phase (Fig. 1a, b). While the listing of some individual genes in more than one pathway (Table 1) likely reflects areas of signaling/pathway cross talk (as these pathways are known to cross talk) and makes it more difficult to fully separate the processes in only 3D, additional visual angles (Supplementary Fig. S2) show clustering of the apoptosis genes.

**Table 1:** List of genes assigned to different groups based on their pathway membership as annotated in Gene Ontology

Gene	Apoptosis (GO:0006915)	Autophagy (GO:0006914)	UPR (GO:0006986)
ATF6	ATF6		ATF6
DDIT3	DDIT3		DDIT3
EIF2AK3	EIF2AK3		EIF2AK3
ATF4	ATF4		ATF4
EIF2A	EIF2A		
HSP90B1	HSP90B1		HSP90B1
HSPA5	HSPA5		HSPA5
XBP1	XBP1	XBP1	XBP1
AMBRA1	AMBRA1	AMBRA1	
ATG12		ATG12	
ATG5	ATG5	ATG5	
ATG7	ATG7	ATG7	
BCL2L1	BCL2L1		
BCL2L2	BCL2L2		
BECN1	BECN1	BECN1	
MCL1	MCL1	MCL1	
MTOR	MTOR	MTOR	
TSC2	TSC2	TSC2	
BCL2	BCL2	BCL2	
SQSTM1	SQSTM1	SQSTM1	
TSC1		TSC1	

Genes from two independent gene expression microarray data sets^21, 22^ were acquired and analyzed as described in the Materials and methods and Supplementary Table S2, and a gene signature of 21 genes was selected. The GO terms for Autophagy Gene Ontology GO:0006914, Apoptosis Process GO:0006915 and Response to unfolded protein GO:0006986.

### Involution progress is defined by changes in tissue architecture, apoptosis, and macrophage infiltration

**Fig. 2:**
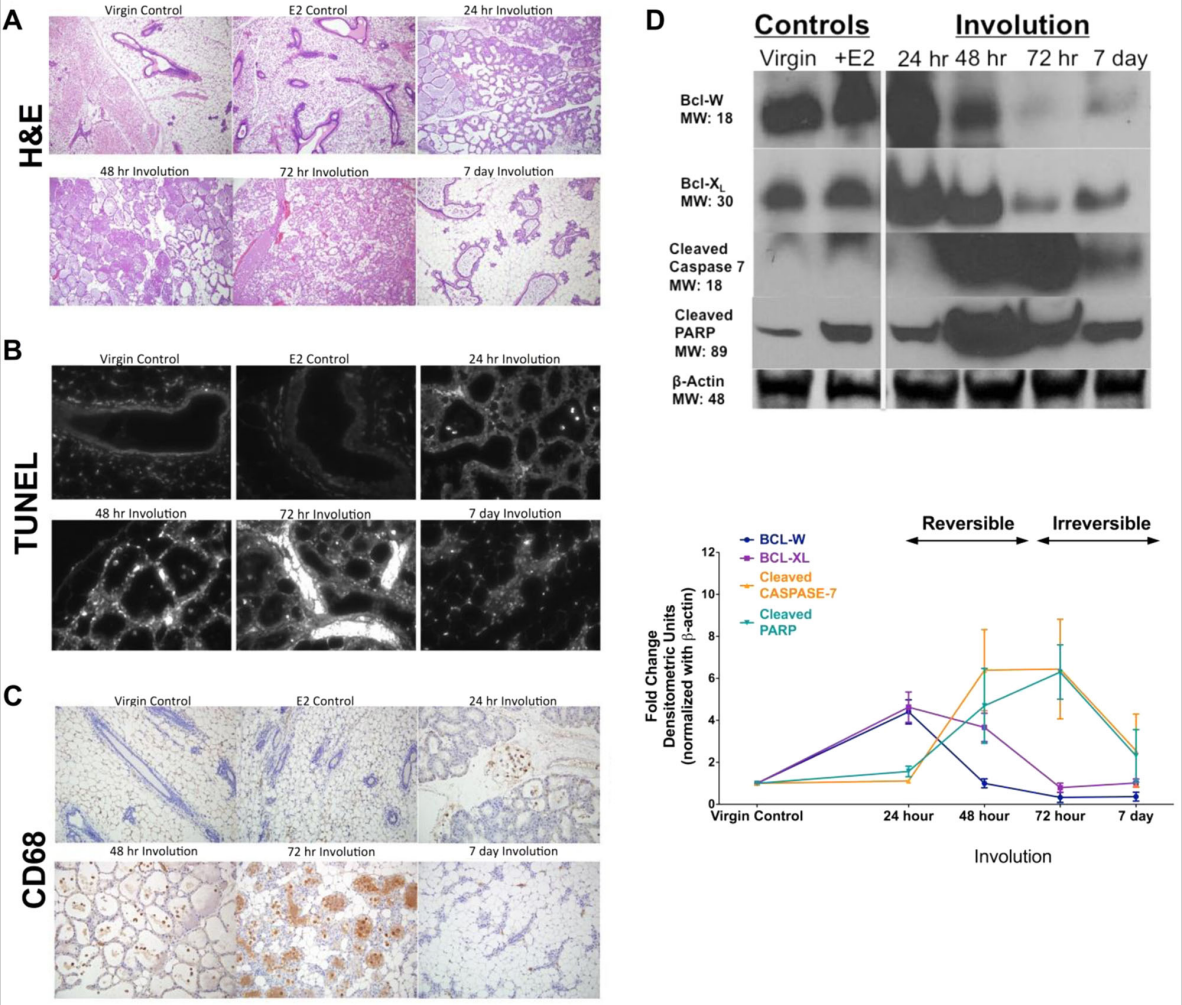
Progression of mammary gland involution based on changes in tissue architecture, apoptosis, infiltrating magrophages, and the expression of apoptosis proteins. Involution time course was created and samples collected as described in the Materials and methods. Mammary glands of virgin mice ± 17β-estradiol (E2) were used as negative controls; E2 treatment was used to block apoptosis. **a** Progression of involution was assessed based on tissue histology in the H&E stained slides and quantified in early involution (24–72 h) by grading of the H&E slides (shown in Supplementary Fig. S1) and in late involution (96 h, 7 d) by using ImageJ analysis of epithelial/adipose tissue area of the H&E stained slides (see Materials and methods); all slides were photographed using Olympus BX 61 microscope and 10× magnification (A–C). **b** Detection of apoptotic cells by TUNEL IHC. **c** Macrophage infiltration visualized by CD68 staining (IHC). **d** Expression of the known apoptosis markers and regulators BCL-W, BCL-XL, Cleaved caspase-7, Cleaved PARP proteins were measured by Western analysis. Results at each time point show average ± SD, *n* = 3

To explore the extent of apoptosis and the possible contribution of UPR and autophagy during involution, we conducted a time-course experiment where involution was initiated by forced weaning after 10 days (peak) of lactation. Samples were collected at 0–96 h, and 7 d. We first established a timeframe of involution progression for our mouse model (Fig. 2a and Supplementary Fig. S1). We then established a quantitative grading for the early involution samples to accurately stage progression (24–72 h; Fig. S1), and used ImageJ quantification of the epithelial/adipose tissue area in the late involution glands (96 h, 7 d). Concurrently, the appearance of apoptotic cells was visualized by terminal deoxynucleotidyl transferase dUTP nick end labeling immunohistochemistry (TUNEL IHC) (Fig. 2b). Adult virgin mammary glands and those from estradiol (E2) treated mice were used as negative controls. E2 treatment was used to inhibit apoptosis, which occurs at low levels in the normal mammary glands of estrus-cycling mice. As shown in Fig. 2b, significantly increased TUNEL staining was detected at 48–72 h of involution, indicating a potent induction of apoptosis. An increase in CD68 staining (Fig. 2c) coincides with increased TUNEL staining, showing that macrophages accompany the increasing number of dying cells in the alveolar lumens. Expression of proteins known to regulate and execute apoptosis (BCL-W, BCL-XL, Cleaved caspase-7, cleaved PARP; Fig. 2d) also showed a temporal pattern. Expression of BCL-family members peaks early (at 24 h), whereas that of caspases peak after onset of the irreversible phase of involution (at 48–72 h; Fig. 2d).

### Autophagy, UPR, and apoptosis signaling components have a distinct temporal pattern during involution

**Fig. 3:**
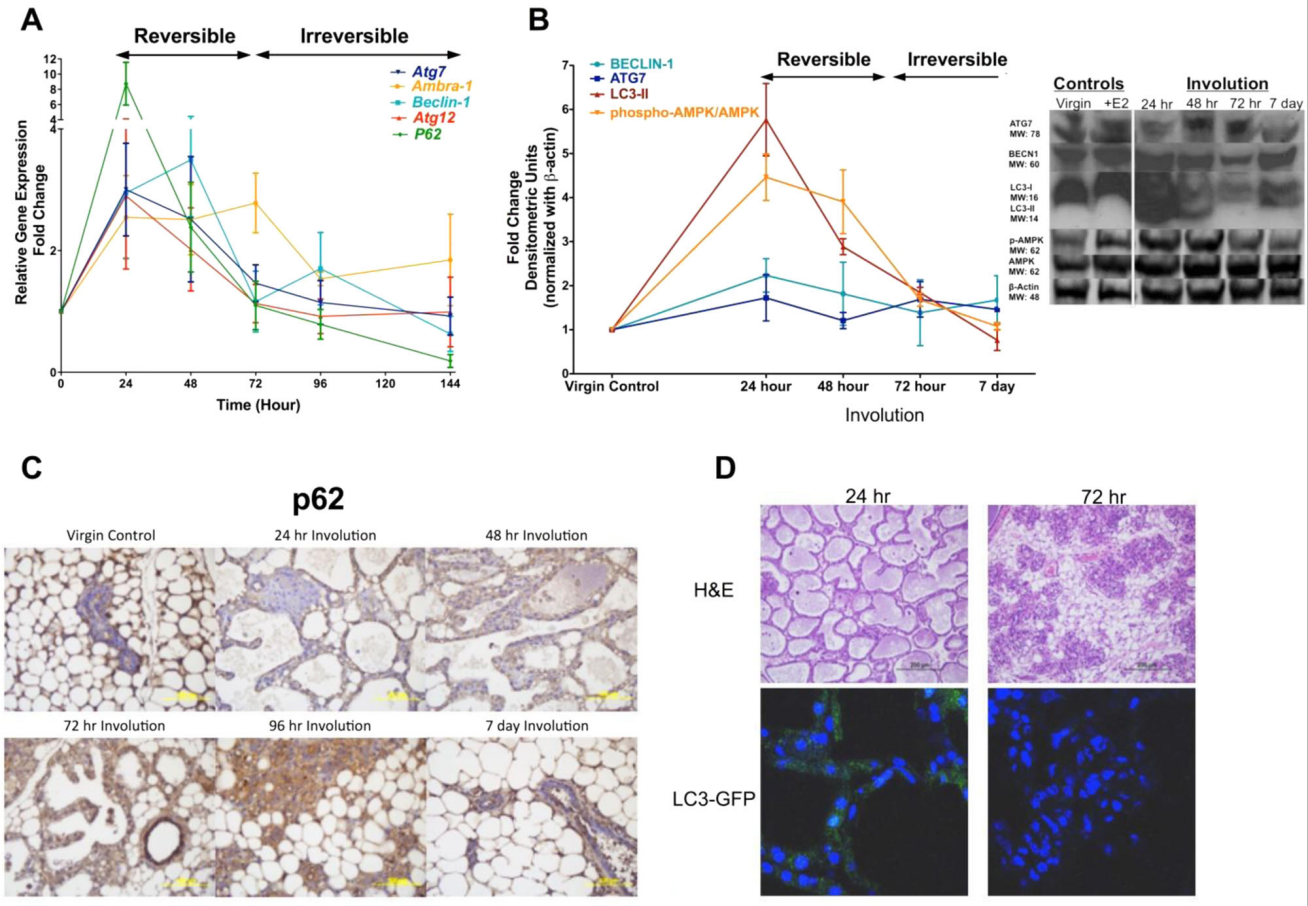
Evaluation of autophagy in the mammary gland involution. **a** Expression of the autophagy genes *Atg7*, *Atg12*, *Ambra-1*, *Beclin-1*, and *p62* was analyzed by qRT-PCR. Lactating mammary glands (involution 0 h) were used as controls; the expression of each gene at each time point is presented as fold-change relative to control. **b** Western analysis of the autophagy proteins BECLIN-1, ATG7, LC3-II, and pAMPK are shown. The horizontal black bars/arrows show the involution switch from reversible to irreversible stage, which has occurred by 72 h after forced weaning (A, B). The vertical bar indicates the involution 0 h (B, Western blot inset). Results at each time point show average ± SD, *n* = 3. **c** Autophagy marker, (downregulation of) p62 specific staining (IHC) at different time points during involution and in virgin control mammary glands. **d** H&E staining (upper panel) and autophagy specific LC3-GFP punctate formation (lower panel) in identical involution samples from LC3-GFP transgenic mice. All slides were photographed using Olympus BX 61 microscope and 10× magnification (C, D)

We next investigated the expression of autophagy regulators relative to the progress of involution and apoptosis. As shown by quantitative reverse transcription polymerase chain reaction (qRT-PCR) (Fig. 3a) and/or Western (Fig. 3b) analysis, during the first phase of involution autophagy markers (low p62; high LC3-II) coincide with peak expression of the known autophagy regulators *Beclin-1*, *Atg7*, *Atg12*, and pAMPK. The expression of Autophagy/Beclin-1 Regulator *Ambra* peaks later, at 72 h (Fig. 3a). IHC data for p62 specific staining (Fig. 3c) and autophagy specific LC3-GFP punctate formation in identical involution samples from LC3-GFP transgenic mice (Fig. 3d) are in accordance with p62 gene and LC3-II protein-expression quantifications.

**Fig. 4:**
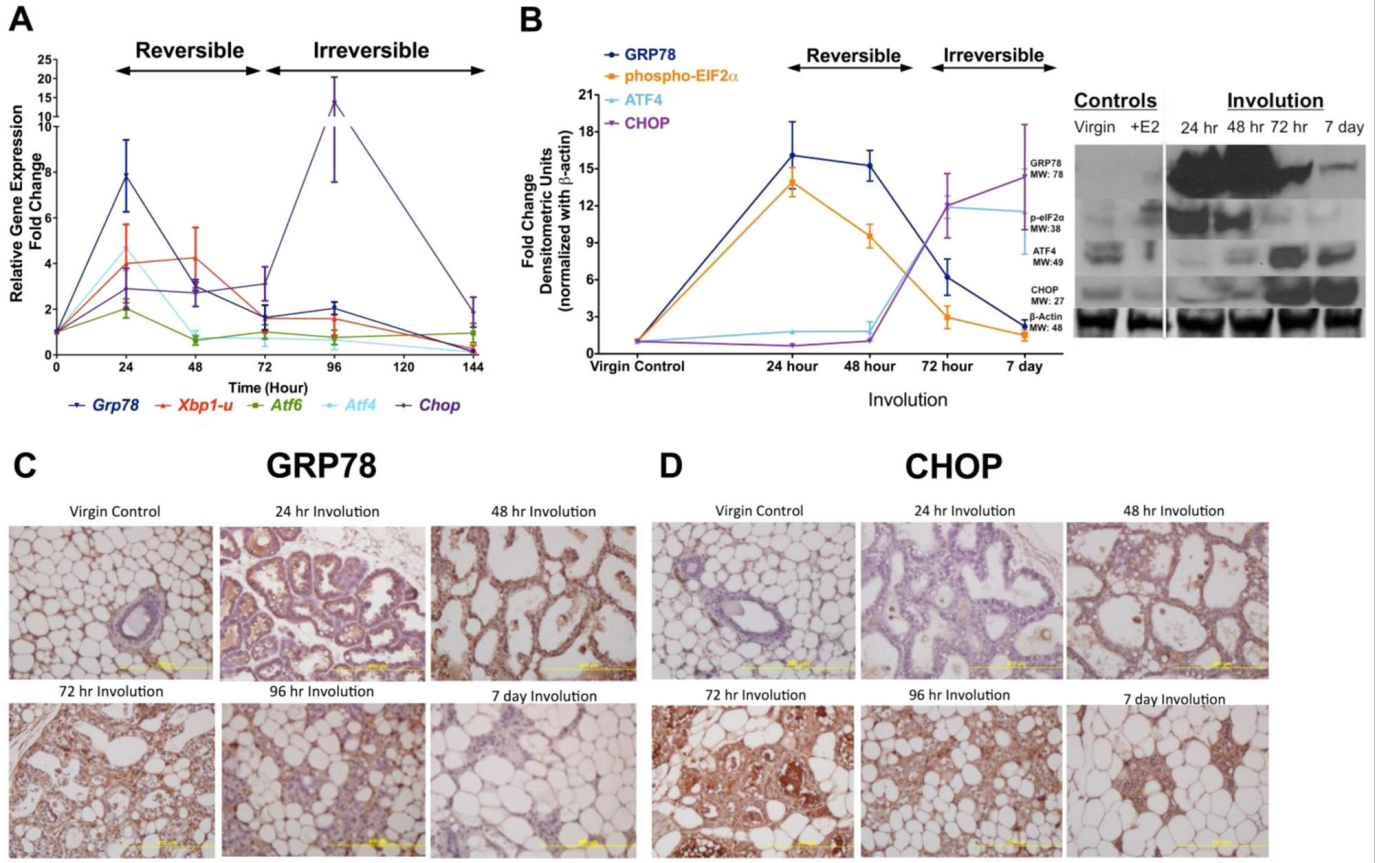
Evaluation of UPR in the mammary gland involution. **a** Expression of UPR genes *Grp78*, *Atf4*, *Atf6*, *Xbp1* (unspliced form), and *Chop*/*Ddit3* was analyzed by qRT-PCR. Lactating mammary gland (involution 0 h) was used as a control, and the expression of each gene at each time point is presented as fold-change relative to control. The horizontal black bar/arrow shows the involution switch from reversible to irreversible stage, which has occurred by 72 h after forced weaning (A, B). **b** Western analysis of UPR proteins GRP78, ATF4, ATF6, XBP1 (unspliced form), phospho-eIF2a, and CHOP/DDIT3. The vertical bar indicates the involution 0 h (B, Western blot inset). Results at each time point show average ± SD, *n* = 3. **c** GRP78 and **d** CHOP/DDIT3-specific staining (IHC) at different time points during involution and in virgin control mammary glands. All slides were photographed using Olympus BX 61 microscope and 10× magnification (C, D)

To verify further the temporal regulation of involution we measured expression of the known upstream regulators of autophagy, the UPR genes *Grp78*, *Atf4,* and *Atf*6, *Xbp1* (unspliced form), phospho-*eIF2a*, and *Chop/Ddit3*. As shown by qRT-PCR analysis (Fig. 4a), expression of the *Grp78*, *Atf4*, *Atf6*, and *Xbp1* mRNAs peak during the first phase of involution (24–48 h), while *Chop/Ddit3* mRNA expression peaks during the second phase (96 h). Protein expression by Western analysis (Fig. 4b) shows the highest protein level of GRP78 and phospho-eIF2a at 24 h, while ATF4 and CHOP/DDIT3 expression increase later (at 72 h and 72 h–7 d, respectively). Analysis of the published data sets^21,22^ showed both *Xbp1* and *Rela* mRNA expression to be elevated during involution, compared with virgin, pregnant, and lactating mammary glands (Supplementary Fig. S3). IHC analyses show an early expression of GRP78 (Fig. 4c), while strong CHOP/DDIT3-specific staining is apparent at later time points (Fig. 4d). Taken together, the time-course analyses of UPR mRNA and protein expression during mammary gland involution show that their expression is tightly orchestrated, occurring concurrent with the expression of autophagy genes and prior to expression of caspases and cleaved PARP, hallmarks of the execution of apoptotic cell death.

### Autophagy and apoptosis are co-regulated during involution

**Fig. 5:**
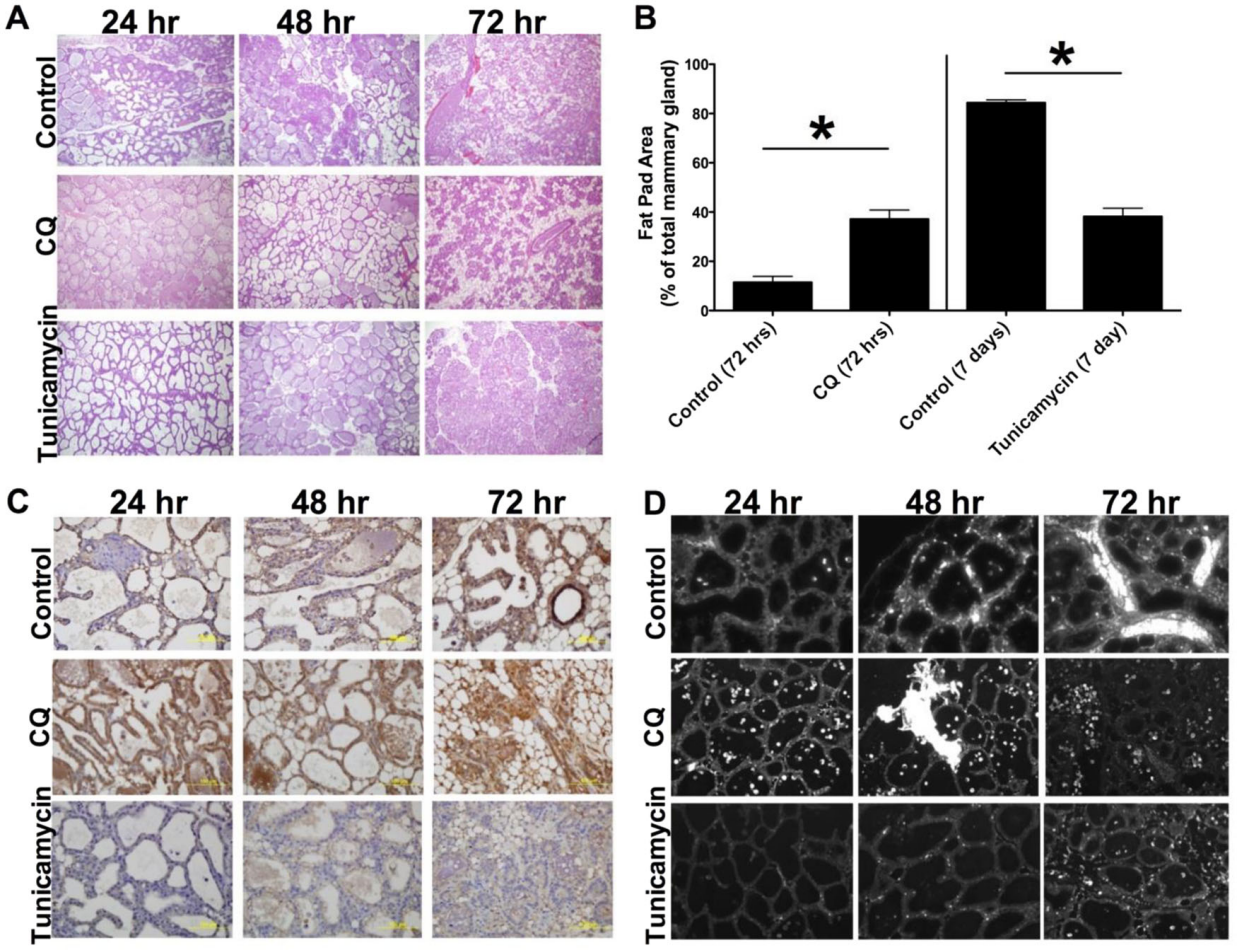
Drug interventions inhibiting and stimulating autophagy enhanced and delayed involution, respectively. At the time of forced weaning (involution 0 h) drug treatments were started to inhibit (with low dose chloroquine [CQ], middle panels) and stimulate (with low dose tunicamycin [Tm], bottom panels) autophagy, as described in the Materials and methods. Time-course involution samples were collected as in Figs. 2–4. **a**, **b** Histology and quantification of involution progress by ImageJ analysis of epithelial/fat pad area of the H&E stained mammary gland slides, as described in Materials and methods. Average ± SD are shown, *n* = 3. Tm vs. vehicle control: Student’s *t* test, *P* ≤ 0.001 (96 h); Mann–Whitney rank sum test *P* ≤ 0.001 (7d). CQ vs. vehicle control: Mann–Whitney rank sum test *P* ≤ 0.001 (72 h). **c** Representative slides of (**c**) autophagy marker (downregulation of) p62 specific staining (IHC), and **d** apoptosis marker TUNEL IHC are shown

To confirm a mechanistic relationship for the link between autophagy and apoptosis during involution, we performed drug intervention studies to either inhibit (daily chloroquine treatment) or stimulate autophagy (daily tunicamycin treatment). We recorded changes in the progress of involution relative to vehicle treated mice, based on grading of mammary gland histology (Fig. 5a) and quantification of epithelium/adipose tissue area (Fig. 5b), autophagy (p62 IHC, Fig. 5c), and apoptosis (TUNEL IHC, Fig. 5d). Results establish that low dose tunicamycin (Tm), a commonly used pharmacologic inducer of the canonical EnR stress response, significantly delayed the appearance of, and decreased the number of, apoptotic cells (Fig. 5a, c, d bottom panels). In contrast, inhibiting autophagy with chloroquine (CQ) administration produced an enhanced onset of apoptosis and advanced initiation of the irreversible phase of involution (Fig. 5a, c, d middle panels). Quantification of changes in mammary gland histology in the Tm and CQ treatment groups is shown as fold-change relative to control (vehicle treated mice; Fig. 5b). Taken together, the results show that autophagy is critical for regulating the progress of involution during its transition from the reversible to the irreversible phase.

**Fig. 6:**
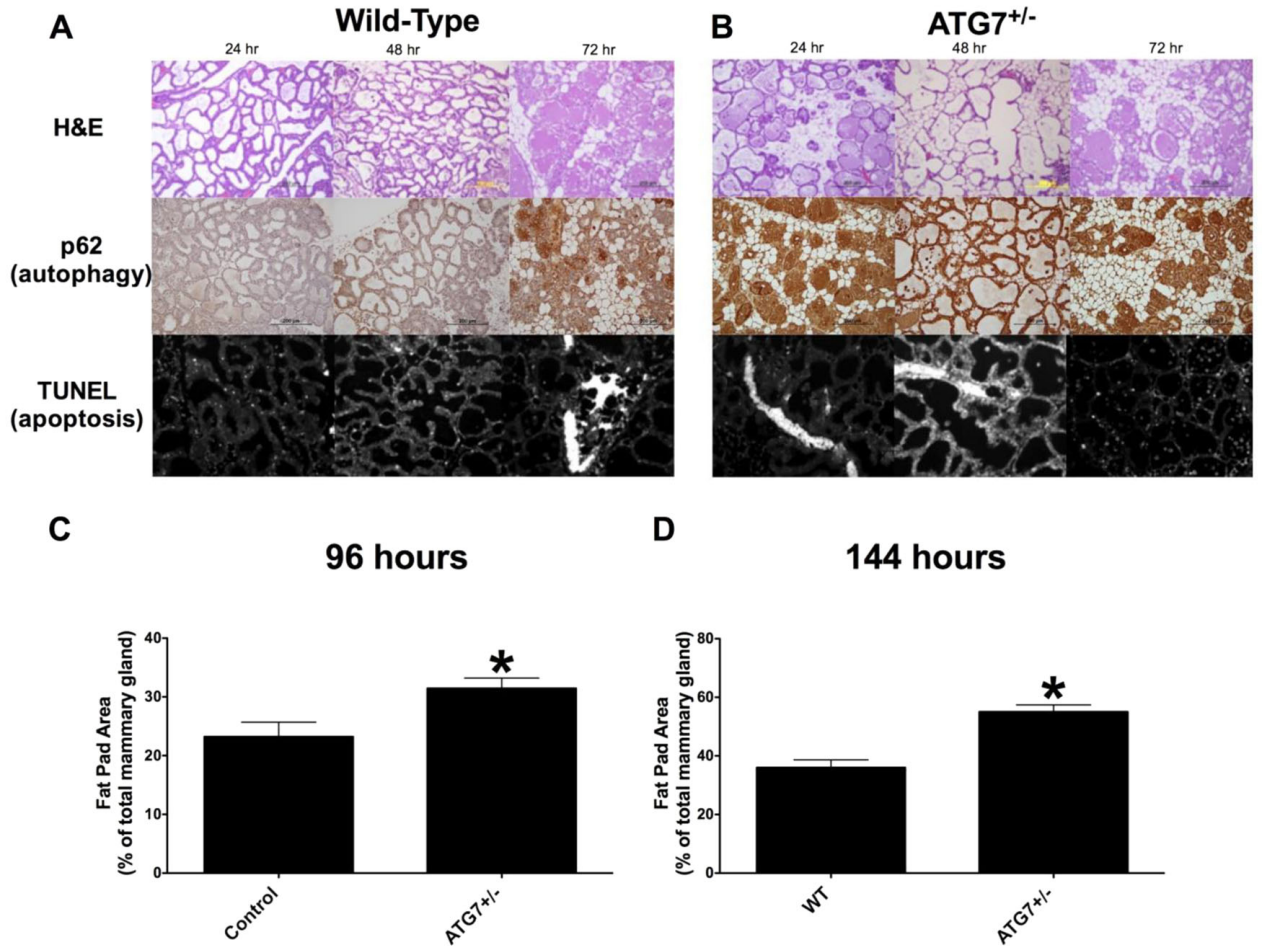
Enhanced involution in autophagy gene deficient mice. An involution time course was created using wild type (Atg7^+/+^) and Atg7 heterozygous (Atg7^+/−^) mice. Samples were collected as in Figs. 2–4 and as described in the Materials and methods. **a**, **b** H&E staining (top panels), p62 IHC (middle panels), and TUNEL IHC (bottom panels) of mammary glands of Atg7^+/+^ and Atg7^+/−^ mice after 24 h, 48 h, and 72 h of involution. **c**, **d** Quantification of epithelial/adipose tissue area of the H&E stained slides as in Fig. 2, and as described in the Materials and methods, and in late involution (96 h, 7 d) by using ImageJ analysis, as described in the Materials and methods. Average ± SD are shown, *n* = 3–5. Student’s *t* test, *P* = 0.026 (96 h), *P* = 0.001 (7 d)

We also created an involution time-course, using an ATG7-deficient (Atg7^+/−^) mouse model^23^, and measured changes in autophagy (p62 IHC, Fig. 6a, b, middle panels) and apoptosis (TUNEL IHC, Fig. 6a, b, bottom panels) in mammary glands during their transition from the first to the second phase of involution (24–72 h). Atg7^+/−^ mice were compared with their Atg7^+/+^ littermate mice, and the experiment was performed as described for the involution models above. Progress of involution was measured by quantification of epithelium/fat pad area in the mammary glands at 96 h and 7 d of involution (Fig. 6c, d). Strikingly, the progress of involution and apoptosis in the mammary glands of Atg7^+/−^ mice, when compared with their Atg7^+/+^ control mice, were enhanced as also seen when chloroquine treated mice where compared with their vehicle treated controls. These data further highlight a critical role for autophagy in the regulation of mammary gland involution.

## Discussion

Pathways that control common house-keeping functions in normal cells are often activated in cancer cells, including breast cancer, a phenomenon termed “nononcogenic addiction”^24^. Several of these processes contribute to normal mammary gland involution including apoptosis, inflammation, and wound healing^2,8,22,25^. Involution is a normal process initiated after pregnancy, without or following an intervening period of lactation (breast feeding), during which the terminally differentiated mammary gland returns to a prepregnancy state resembling the virgin gland. The process involves a complex and robust period of programmed cell death and changes in tissue architecture that include extracellular matrix (ECM) remodeling and adipogenesis^1,6,7^. Reflecting the ability of the normal adult mammary gland to undergo robust de-differentiation and tissue remodeling, mammary involution has been implicated as a driver of ductal carcinoma in situ (DCIS), and may be a key contributor to pregnancy-associated breast cancer^3,4^. In rodent models, multiple studies have shown involution to enhance tumor cell growth, local invasion, and metastasis^5,26^. To date, studies have focused mostly on the role of apoptosis and classical/nonclassical pathways of cell death in mammary involution^27,28^. We now show that the UPR regulates autophagy (macroautophagy), and that both activities are co-regulated with apoptosis during involution. These integrated activities provide a key survival component that regulates transition from the reversible to irreversible phases (“involution switch”) early in the initiation of mammary gland involution. Using normal mouse mammary tissue, we establish that autophagy stimulation with tunicamycin treatment leads to prolonged persistence of secretory mammary epithelium, while inhibition of autophagy with chloroquine or ATG7 haploinsufficiency results in enhanced/precocious involution through enhanced apoptosis.

Comprehensive global gene expression analyses of genes differentially expressed during mammary development have previously been used to identify involution-associated genes that may contribute to breast tumorigenesis^8,21,22,29,30^. Here, we used only data sets that included several involution time points and without pooling tissue samples (C57Bl/6 mice^21^; BALB-c mice^22^). Since involution is triggered by the increased cellular stress that arises from milk accumulation in the secretory epithelium after cessation of nursing^7^, we focused on UPR genes and its downstream signals. The UPR is activated by an inappropriate accumulation of proteins in the endoplasmic reticulum, and we have previously shown how the UPR can activate autophagy^19,31^. In both data sets, we found differential expression of UPR, autophagy, and apoptosis genes when compared with nonpregnant (virgin) mammary glands (Supplementary Table S2). Principal component analysis (PCA) of the differentially expressed genes clearly shows that apoptosis, autophagy, and UPR and its downstream genes were present in separable clusters defined by the top three principal components (Fig. 1a, b; Supplementary Fig. S2). Thus, these genes are expressed in a temporally orchestrated fashion relative to the progress of involution. To validate these observations independently, we applied an established mouse model of forced involution and created a time course of mammary gland samples. Consistent with previous studies, tissue architecture (assessed in histological sections; Fig. 2a, Supplementary Fig. S1) and the extent of apoptosis (Fig. 2b) confirmed the two distinct phases of involution^32–34^. The number of apoptotic cells peaked at the beginning of the irreversible phase of involution (48–72 h), concurrent with increased levels of cleaved caspase-7, and cleaved PARP (Fig. 2d). Increased expression of anti-apoptotic BCL2 family members was detected early, prior to increased TUNEL staining and expression of the proapoptotic proteins. Our results are consistent with observations from Bcl-2 overexpressing and Bcl-XL knockout mice indicating a key protective role for BCL-2 family members in the regulation of involution^35–37^.

Controlled influx of macrophages and other immune cell types, to eliminate the dead and dying mammary cells, is a central component of involution^38,39^. Depletion of macrophages leads to delayed postpartum involution (measured at day 3^39^). We show that increased infiltration of macrophages coincided with the peak in cell death (highest TUNEL positivity) after 48–72 h of involution, reflecting initiation of the irreversible phase of involution (Fig. 2c).

We hypothesized that, through UPR signaling, autophagy could provide a key survival component in the early phase of mammary gland involution, during which tissue architecture is preserved and involution is still reversible. A physiologically critical window would thereby be maintained during which lactation can resume if suckling restarts. Thus, any temporary interruption in lactation would not lead to the end (failure) of nursing and possible death of the offspring. We focused on the signaling events around the “involution switch”, when reversible involution becomes irreversible (after 48 h in mouse), and show an orchestrated regulation of autophagy and UPR genes after 24–96 h of mammary involution initiation. We observed a decline in p62 expression levels (Fig. 3a), upregulation of LC3-II protein expression (Fig. 3b), and increased LC3-GFP punctate formation in IHC (Fig. 3d) at 24–48 h compared with those obtained 72 h postweaning, indicative of activated autophagic flux. Upregulation of phosphorylated AMPK and decreased TORC1 complex (preliminary data) expression show that the promotion of autophagy in the reversible phase of involution might be regulated by mTOR inhibition. p62 protein expression (IHC) in the mammary epithelium was increased up to 96 h after initiation of involution, suggesting a decrease in autophagy during the irreversible phase of mammary gland involution (Fig. 3c). These results appear consistent with earlier observations showing induction of autophagy by the detachment of luminal cells from their extracellular matrix^9^, and autophagy protecting mammary epithelial^9,15^, DCIS^40,41^, and breast cancer cells^10,42^ from anoikis.

Other observations suggest that autophagy can contribute to both prosurvival and prodeath outcomes during mammary involution (refs.^9,15^ vs. ref.^16^). Here, we show that signaling through UPR regulates both functions of autophagy (prosurvival; prodeath), but in a sequential and orchestrated fashion. Results of time course experiments showed elevated *Grp78* (Fig. 4a, b), *Xbp1* (unspliced form; Fig. 4a), and phosphorylated eIF2α (Fig. 4b) expression 24–48 h postweaning, the time during which involution is reversed if suckling resumes. However, stimulation of *Atf4* and *Chop/Ddit3* occurred later, at 72–168 h of involution (Fig. 4a, b). Overexpression of ATF4 can lead to impaired lactation and accelerated involution marked by increased apoptosis, suggesting that increased ATF4 promotes cell death in the mammary gland^43^. ATF4 stimulates the proapoptotic protein, CHOP/DDIT3^44^, and we found that CHOP/DDIT3 expression increased 48–168h after forced weaning and correlated with increased cell death (Fig. 4a, b, d). Our results highlight the timing and duration of UPR-autophagy signaling molecules/pathways contributing both to prosurvival and prodeath outcomes in response to cellular stress in normal mammary tissue.

To verify a causal relationship between autophagy and apoptosis in the progression of involution, we performed drug intervention experiments to both block (with chloroquine) and enhance (with tunicamycin) autophagy. Dosing lactating mice with chloroquine starting at the time of forced weaning accelerated involution (as determined by H&E, Fig. 5a middle panel, Fig. 5b) and apoptosis (Fig. 5d middle panel), implying that autophagy promotes survival and is a vital signaling component of the reversible phase of involution. The increase in p62 expression confirms the inhibition of autophagy by chloroquine (Fig. 5c middle panel). The opposing intervention, where lactating mice are treated with tunicamycin starting at the time of forced weaning, reduced, and/or delayed apoptosis (Fig. 5d, bottom panel) and sustained autophagy, seen as low expression of p62 (Fig. 5c bottom panel). Thus, prolonged autophagy can extend the reversible phase of mammary gland involution. These data imply a possible translational application of autophagy stimulation to promote and prolong lactation in women with difficulties or irregularities in breast feeding, especially in developing countries where mother’s milk production provides the primary source of babies’ nutrition. In contrast, chloroquine administration likely has an adverse effect on some nursing women in those countries where this drug is used for malaria prevention/treatment.

We also performed an involution time course study using *Atg7* heterozygous (^+/−^) and wild-type (^+/+^) mice^23^ to confirm further the causal relationship between intact autophagy signaling and apoptosis in the progression of involution. As with chloroquine treated mice, *Atg7*^+/−^ female mice exhibited an enhanced mammary involution compared with wild-type mice (Fig. 6a, b; quantification Fig. 6c, d), characterized by an early/precocious induction of apoptosis (Fig. 6a, b, bottom panels). Since ATG7 is also required for efferocytosis^9,16,45^, the engulfment and phagocytosis of dead cells and apoptotic bodies by viable mammary epithelium^45,46^, delayed involution was reported in epithelial *Atg7*-deficient mammary glands^16^. In contrast, we found accelerated involution in *Atg7*^+/−^ mouse mammary glands. However, Teplova et al. used a mouse model where most mammary epithelial cells, but not the mammary stromal cells, were ATG7 deficient. Both cell compartments are ATG7 deficient in the mouse model used in the present study. As indicated by Castello-Cros et al.^47^. and Sanchez et al.^10^, stromal cells are of major importance because they use autophagy to support the survival of epithelial cells. Consistent with our results, Debnath et al.^9^. reported that depletion of either ATG5, ATG6, or ATG7 inhibited autophagy and enhanced luminal apoptosis, concluding that autophagy promotes mammary epithelial cell survival during anoikis. ECM detachment-induced autophagy occurs even in the absence of apoptosis in Bcl2 overexpressing cells. Thus, the contributions of autophagy to cell survival during ECM detachment are independent of the cells’ apoptosis competency^9^.

In conclusion, we have established an integral role for the temporally orchestrated expression of key UPR and autophagy signaling molecules in mammary gland involution during the critical transition from a reversible to irreversible phase of tissue regression/remodeling. Confirming the causal relationship, inhibition of autophagy by either drug treatment or ATG7 haploinsufficiency enhances epithelial cell death and advances involution. Conversely, stimulation of autophagy delays robust cell death and prolongs the reversible phase of mammary gland involution. To our knowledge, our results are the first preclinical in vivo data supporting the hypothesis that autophagy may promote the survival of breast cells lacking an appropriate matrix contact in DCIS lesions and/or disseminating tumor cells. The clinical value of this hypothesis is currently being tested in the PINC trial (“Preventing Invasive Breast Neoplasia with Chloroquine” [clinicaltrials.gov/show/NCT01023477]^40^), which is assessing the efficacy of neoadjuvant anti-autophagy therapy in inhibiting the progression of DCIS to invasive disease such as invasive ductal carcinoma.

## Materials and methods

### Materials

Antibodies were purchased from the following vendors: GRP78 (Western blots), CHOP/DDIT3 (Western blot), phospho-eIF2α, Beclin-1, Atg7, LC3B, Bcl-W, Bcl-XL, Cleaved caspase-7 and PARP (Cell Signaling Technology), GRP78 (IHC), ATF4, β-Actin and horseradish peroxidase (HRP)-secondary antibodies (Santa Cruz Biotechnology), CHOP/DDIT3 (IHC) (Abcam). Apoptosis was measured by TUNEL staining using a fluorescein-based in situ cell death detection kit (Roche).

### Murine mammary gland involution model

All animal procedures were approved by the Georgetown University Animal Care and Use Committee and performed following the National Institutes of Health guidelines for the proper and humane use of vertebrate animals in biomedical research. Mice were housed in a temperature- and humidity-controlled room under a 12-h light–dark cycle. Female ATG7 wild type (^+/+^), heterozygous (^+/−^; Tokyo Metropolitan Institute of Medical Sciences, Japan^23^,) and LC3-GFP mice (obtained through NIH from RIKEN BioResource Center^48^) and C57Bl/6 mice (Harlan, USA) were mated to produce and nurse pups for approximately 10 days before forced weaning to induce involution (Inv. 0 h); litters were harmonized to contain six to eight pups. At the end of each experiment, animals were euthanized and mammary glands were collected at necropsy 24, 48, 72, 96 h, and 7 d after forced weaning. Abdominal #4 glands (from the same animal) were snap frozen and used later for gene expression analyses by qRT-PCR. Thoracic #2–3 glands were snap frozen for protein analyses by Western and fixed in formalin for immunohistochemical analyses. 3-5 mice were used at each time point. PCR primers for murine ATG7 and LC3 are given in Supplementary Table S1A.

### Intervention studies

As with the above involution model, C57Bl/6 mouse dams were allowed to nurse litters of six to eight pups for 10 days before forced weaning (Inv. 0 h), at which time point either chloroquine (CQ; Sigma-Aldrich, USA), tunicamycin (Tm; Calbiochem EMB Bioscience Inc.) or vehicle treatments were initiated. CQ was given in drinking water (0.24 mg/ml, resulting in ~1 mg daily dose per mouse^49^). Tunicamycin was injected *i.p*. (63 ng per mouse^50^) once daily until the mice were euthanized. Mammary glands were collected after 0, 24, 48, and 72 h as above. At each time point, three to five mice were included and the glands were collected as indicated above.

### Quantification of involution progression

Ten fields in each hematoxylin and eosin (H&E) stained slide of a thoracic #3 mammary gland were systematically photographed using an Olympus BX 61 microscope and 10× magnification at each involution time point, treatment, and for each genotype. Ten fields (H&E stained slides, 10× magnification) in each mammary gland of 24, 48, and 72 h involution time points were graded, adapted with permission from Pai and Horseman^51^, in stages 1–8 as shown in Supplementary Fig. S1. Results are given as fold-change relative to the appropriate vehicle control gland at each time point and treatment. For the 96 h and 7 d involution time points, the percentage of epithelium/adipose tissue per total mammary gland area in the H&E stained slides (ten fields of each slide, 10× magnification) was measured using ImageJ software [http://rsbweb.nih.gov/ij/] according to the IJ 1.46r user guide. *Atg7*^+/−^ mouse mammary glands were compared with *Atg7*^+/+^ glands at each time point. Statistical analyses (*t* test; Mann–Whitney rank sum test) were performed using Sigma Stat 3.0.

### PCA and correlation analysis

The data sets published by Clarkson et al.^21^ and Stein et al.^22^ were acquired by following the data collection method in Zhao et al.^52^. Data normalization was performed using the Plier method in Affymetrix expression console; log2 transformation was made subsequently. A gene signature of 21 genes was selected (Supplementary Table S2) from the QIAGEN PCR Array list to represent autophagy, UPR (downstream) UPR (internal), and apoptosis. Assignement of these genes to different groups was based on their pathway membership as annotated in GO. In the human genes feature using the GO terms for Autophagy Gene Ontology GO:0006914, Apoptosis Process GO:0006915 and Response to unfolded protein GO:0006986, only 5/21 of the genes we detected belong uniquely to a single GO term (Table 1). Thus, since many genes are shared between these pathways, we did PCA (Fig. 1a, b, Supplementary Fig. S2) on each pathway respectively and made a one-to-one gene—pathway mapping based on the importance of a gene in their corresponding pathway.

In addition, in order to investigate the relationship between autophagy and UPR during the involution stage, we selected *n* = 4 genes/group (Table 1, Supplementary Table S2) and calculated the correlation coefficients of all possible permutation among the genes representing autophagy and UPR (UPR was combined). In both datasets, the top ten combinations all has a correlation coefficient >0.95.

### Quantitative real-time reverse transcription PCR

Total RNA was extracted from frozen mammary gland samples by the trizol method. 1 µg RNA was used for cDNA generation using the Biorad iScript cDNA synthesis kit. 1/400 of the cDNA products were used in each 20 μl qRT-PCR reaction to detect mRNA levels. The reference gene used for probe normalization in the qRT-PCR was β-Actin (Actβ). Primers used in the reaction were summarized in Supplementary Table S1B. Primers for murine *Ambra1*, *Atf4*, *Atf6*, *Atg12*, *Atg7*, *Becn1*, *p62*, *Chop*/*Ddit3*, *Grp78*, *Grp94*, and *Xbp1* (unspliced) were designed to give <150-base pair products. Primer specificity was confirmed by gel electrophoresis (produce a single band). Reactions were performed using Biorad SYBR Green Fast qRT-PCR mix and the ABI real-time PCR detection system. Relative mRNA levels were calculated using the comparative Ct method (ΔCt^53^).

### Western blot hybridization

Mammary glands were solubilized by sonication in RIPA lysis buffer (50 mM Tris-HCl pH 7.4, 150 mM NaCl, 1% NP40, 0.25% Na-deoxycholate, 1 mM PMSF, 1 mM sodium orthovanadate, 1× Roche complete mini protease inhibitor cocktail). Protein concentration was determined using a standard bicinchoninic acid assay. Proteins were size fractionated by polyacrylamide gel electrophoresis and then transferred to a nitrocellulose membrane. Nonspecific binding was blocked by incubation with Blotto (tris-buffered saline with 5% powdered milk and 1% Triton X-100) for 1 h at room temperature. Membranes were incubated overnight at 4 °C with primary antibodies, followed by incubation with polyclonal HRP-conjugated secondary antibodies (1:2000) for 1 h at room temperature. Immunoreactive products were visualized by chemiluminescence (SuperSignal Femto West, Pierce Biotechnology, Rockford, IL) and quantified by densitometry using the ImageJ digital densitometry software (http://rsbweb.nih.gov/ij/) with β-Actin and Ponceau-S staining as loading control.

### Tissue staining and IHC

Mammary glands were fixed in 10% formalin for 24 h prior to embedding in paraffin. Embedded tissues were cut into 5 µm thick sections and stained with H&E to determine morphology. Apoptosis was visualized by TUNEL staining. Briefly, mammary gland tissue sections were examined for apoptosis using fluorescein dUTP nick end labeling. Positive- and negative-control slides provided with the kit were used in each assay to ensure consistency. Immunostaining was performed with an antibody to CD68 (1:100), p62 (1:100), CHOP/DDIT3 (1:100), GRP78 (1:100) using the streptavidin–biotin method. Stained sections were visualized and photographed. GFP fluorescence was detected from samples fixed in 4% paraformaldehyde–PBS overnight, followed by immersion in 20% sucrose/PBS at 4 °C for 24 h and cryoprotection in 30% sucrose/PBS at 4 °C until they sunk. Samples were embedded in optimal cutting temperature compound, frozen, and cut in slides using routine procedures.

### Statistical analyses

All statistical analyses (Student’s *t* test; Mann–Whitney rank sum test) were performed using Sigma Stat 3.0, and detailed in the text where applicable.

## Electronic supplementary material

Supplementary figure legends

Supplementary Figure S1

Supplementary Figure S2-S3

Supplementary Table S1

Supplementary Table S2
