# Supplementary material for: Autophagy and unfolded protein response (UPR) regulate mammary gland involution by restraining apoptosis-driven irreversible changes
Source: Cell Death Discov. 2018 Oct 15;4:40. doi: 10.1038/s41420-018-0105-y (PMC6186758; doi:10.1038/s41420-018-0105-y)
Supplement: Supplementary file 1 — Supplementary figure legends [file 41420_2018_105_MOESM1_ESM.docx]

Supplementary Tables and Figure Legends:

**Table S1.** **Primer sequences** for Atg7 and LC3 mouse genotyping (**A**) and for quantitative real time PCR (**B**) used in the study.

**A.**

| **Gene** | **Primer name** |
| --- | --- |
| Atg7 | **Atg7 IN13-F1** GCACTCTTACCTGAATGGCTGAG  **Atg7 IN14-R1** CAGTGGCCAGCCATTTCCAGC  **Atg7 IN13-R4** GCAAGCTCACTAGGCTGCAGAACC |
| LC3  amplifies the third intron of the LC3 genome as an internal control | **GFP1**  5′-TCCTGCTGGAGTTCGTGACCG-3′  **LC3**  5′-TTGCGAATTCTCAGC-CGTCTTCATCTCTCTCGC-3′  **mLC3ex3GT**  5′-TGAGC-GAGCTCATCAAGATAATCAGGT-3′  **mLC3ex4AG**  5′-GTTAGCATT-GAGCTGCAAGCGCCGTCT-3′) |

**B.**

| **Gene** | **Forward sequence** | **Reverse sequence** |
| --- | --- | --- |
| ACTβ | CTGTCCCTGTATGCCTCTG | ATGTCACGCACGATTTCC |
| AMBRA | GGGCAGTAATTGGAGATGGAC | GTACCAGGACATTCACAGAGG |
| ATF4 | ATGGCGTATTAGAGGCAGC | CTTTGTCCGTTACAGCAACAC |
| ATF6 | GAGGCTGGGTTCATAGACATG | GCTAGTGGTTTCTGTGTACTGG |
| ATG12 | ACCATCCAAGGACTCATTGAC | CCATCACTGCCAAAACACTC |
| ATG7 | TCTCCTACTCCAATCCCGTG | TGCTCATGTTGAACCCTCTG |
| BECLIN1 | ACACAGTCCAGAAAAGCTACC | GTACCGACTTGTTCCCTATGG |
| CHOP | TGTTGAAGATGAGCGGGTG | AGGTTCTGCTTTCAGGTGTG |
| CK18 | ACACCAACATCACAAGGCTG | TTCCACAGTCAATCCAGAGC |
| GRP78 | AGTTGATATTGGAGGTGGGC | CATTGAAGTAAGCTGGTACAGTAAC |
| GRP94 | AACCTCTGCTCAACTGGATG | TTGTGCCTTCATGATCCTCTC |
| HPRT | CCTCATGGACTGATTATGGACAG | TCAGCAAAGAACTTATAGCCCC |
| p62 | CCTATACCCACATCTCCCACC | TGTCGTAATTCTTGGTCTGTAGG |
| XBP1 | AAGAAAGCCCGGATGAGC | AGCGTGTTCTTAACTCCTGG |

**Table S2.** **Selected genes representing the processes of apoptosis, autophagy and the unfolded protein response (UPR).** Our prior knowledge of signaling in breast cancer was used to create a list of genes that would likely represent adequately the processes of apoptosis, autophagy and the unfolded protein response. We then validated the selected gene set for this purpose using Gene Ontology Consortium database (GO; <http://www.geneontology.org/page/go-database>) and Fisher’s Exact test.

|  |  |  |  |  |  |  |
| --- | --- | --- | --- | --- | --- | --- |
| **PCR Array** | **Human** | **total** | **expected** | **over/under** | **p-value** | **gene** |
| **Apoptosis** | 384 | 3 | 1.43 | + | 1.12E-03 | BCL2 SQSTM1 TSC1 |
| **Autophagy** | 384 | 10 | 0.22 | + | 3.43E-09 | AMBRA1 ATG7 BCL2L1 BECN1 MCL1 MTOR TSC1 ATG12 ATG5 BCL2L2 |
| **Unfolded Protein Response** | 384 | 8 | 0.08 | + | 1.76E-06 | ATF4 ATF6 DDIT3 EIF2A EIF2AK3 HSP90B1 HSPA5 XBP1 |

**Supplementary Figure Legends:**

**Fig. S1.** **Grading of mouse mammary gland involution from H&E slides.** The most characteristic histologies are pointed out in each stage. Vertical bars indicate stages typically seen relative to involution progress (time; h). Representative sections are shown below, together with the defined stage(s). Dotted red line marks the region of interest (ROI). All slides were photographed using Olympus BX 61 microscope and 10x magnification.

**Fig. S2. Principal Component Analysis (PCA) of gene expression microarray data sets.** Gene selection, analysis and presentation are as described in Fig. 1A,B, and in the Materials and Methods. Additional visual angles showing clustering of the apoptosis genes (blue dots).

**Fig. S3.** ***Xbp1* and *RealA* mRNA expression in virgin, pregnant, lactating and involuting mouse mammary gland in two independent gene expression microarray data sets** ^21, 22^.
