# Supplementary material for: Autophagy and unfolded protein response (UPR) regulate mammary gland involution by restraining apoptosis-driven irreversible changes
Source: Cell Death Discov. 2018 Oct 15;4:40. doi: 10.1038/s41420-018-0105-y (PMC6186758; doi:10.1038/s41420-018-0105-y)

Supplementary Figure S1.

Involution progress

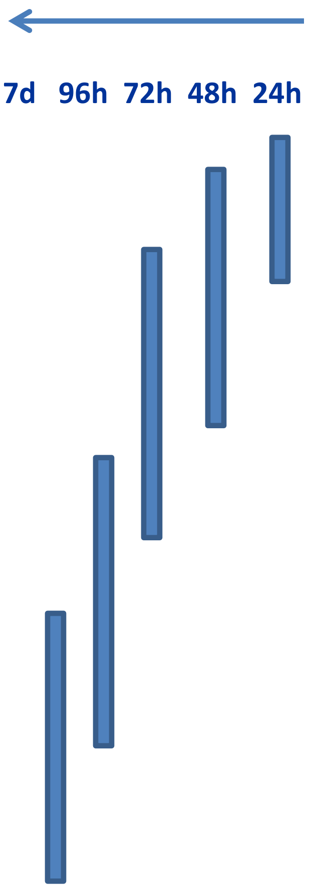

Stage 1: alveoli distended

Stage 2: alveoli secretion filled

Stage 3: alveoli filled with degradation droplets (protein/lipid)

Stage 4: dying cells and macrophages in alveolar and ductal lumen

Stage 5: new adipocytes forming

Stage 6: dilated ducts

Stage 7: alveoli collapsed

Stage 8: total fat pad area > total epithelium area

Stage 1: Distended alveoli due to accumulating milk , only few dying cells

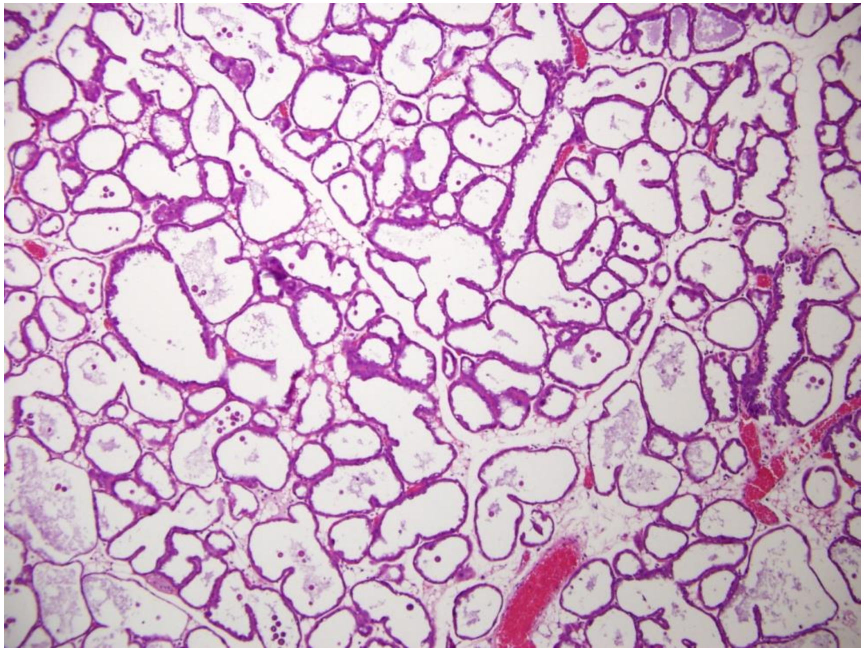

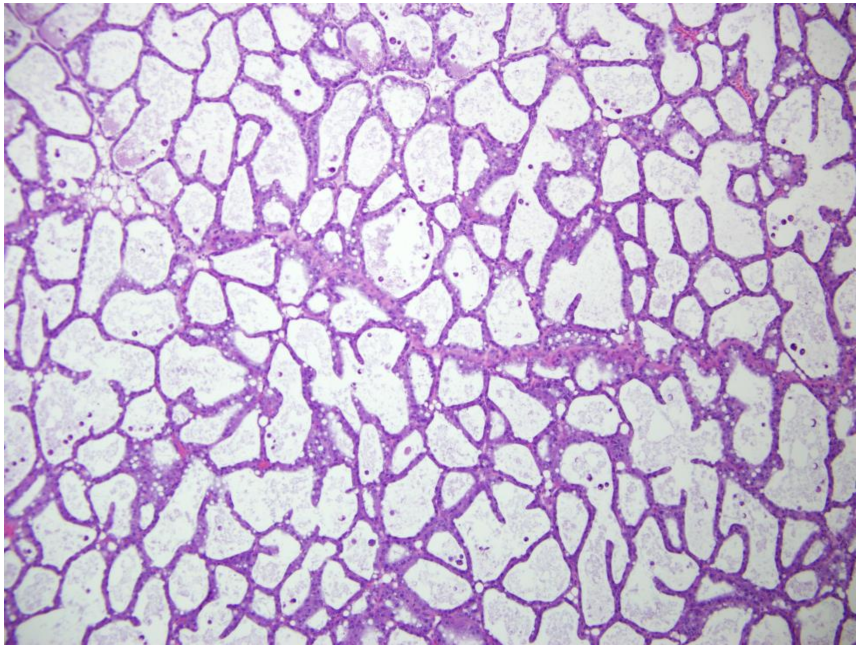

Stage 2: Secretion filled alveoli, few dying cells & macrophages

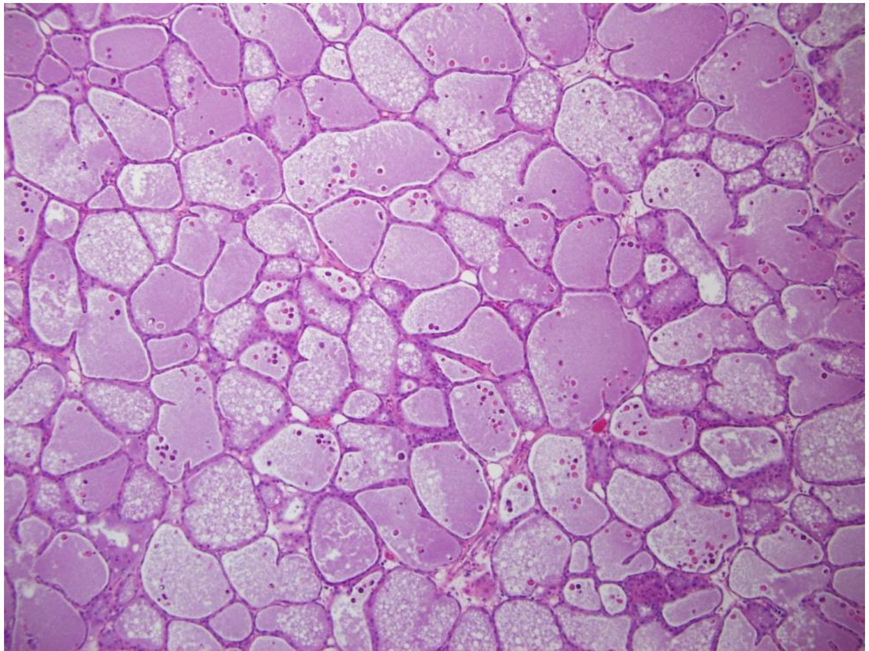

24h involution (vehicle control; 10x)

Stages 1-2

Stage 3: Alveoli filled with degradation droplets

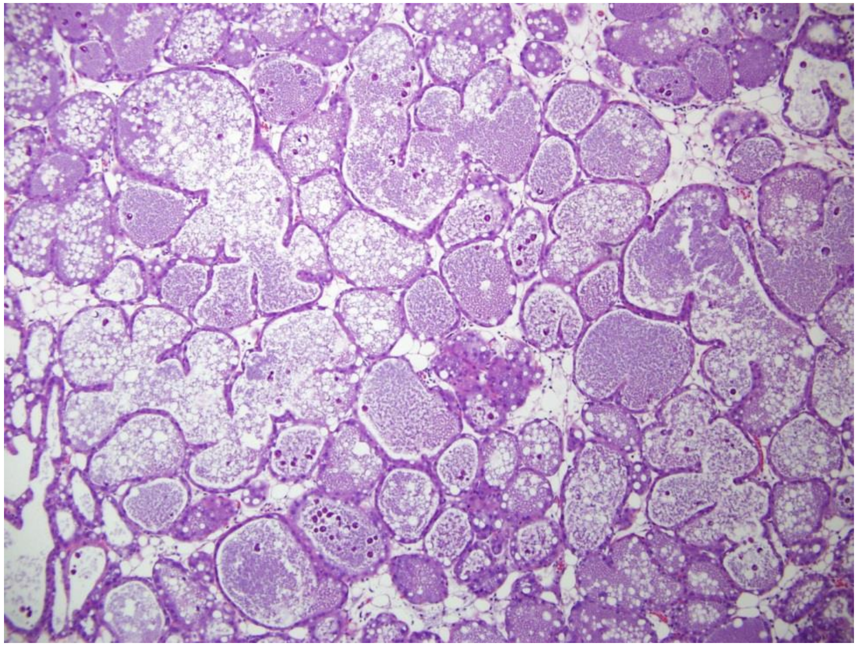

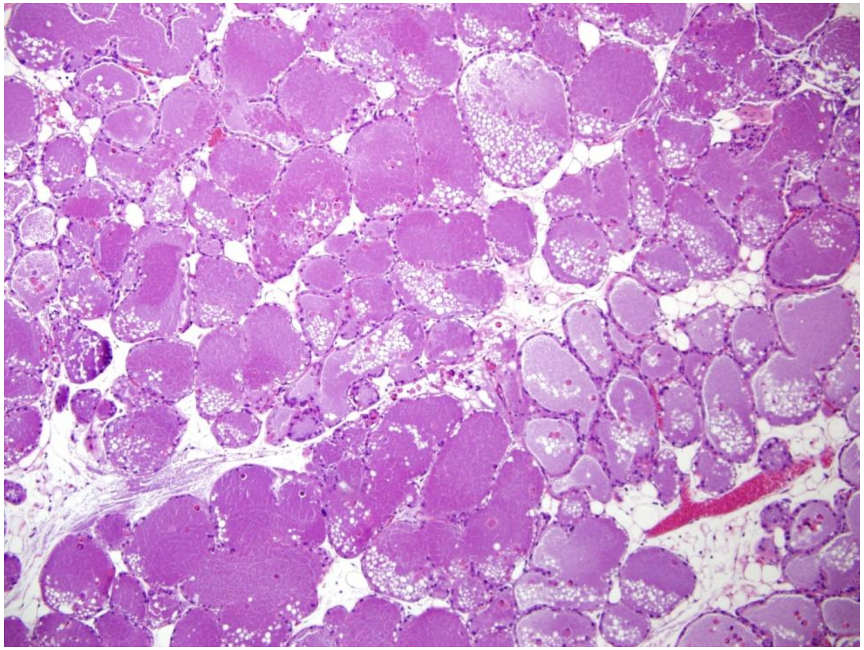

Stage 4: Also plenty of apoptotic (small, compact) cells and macrophages inside the lumen
(*insert*: IHC stain for CD68+ cells, indicating macrophages at 48h of involution; vehicle control, 10x)

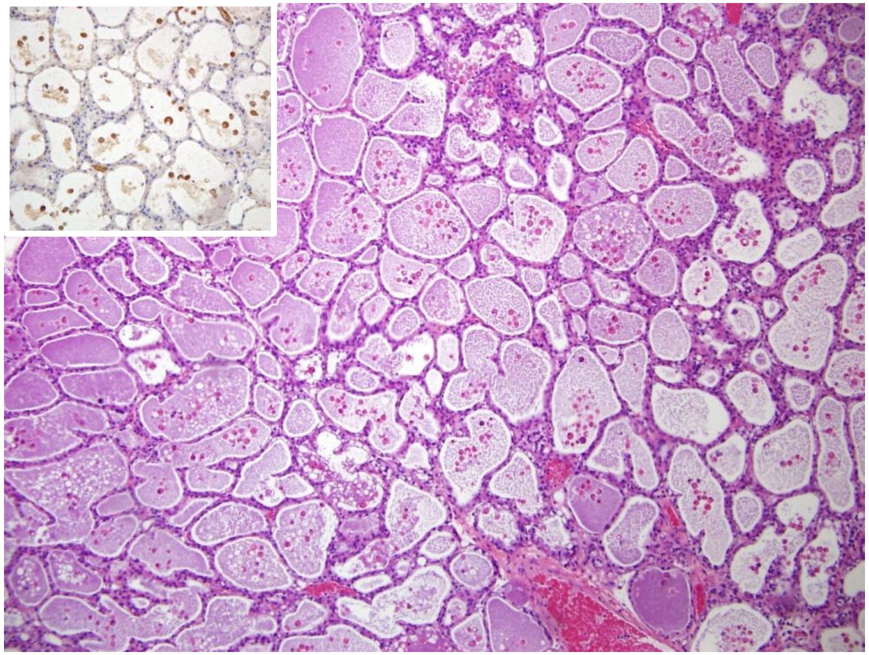

48h involution (vehicle control; 10x)
Stages 2-4

Stage 5: Adipogenesis

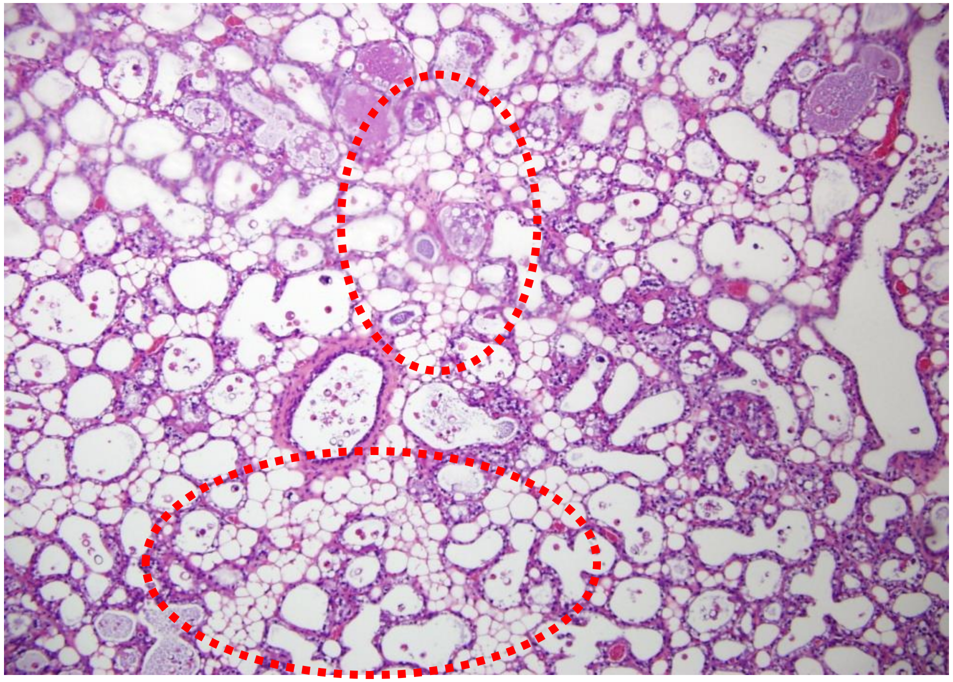

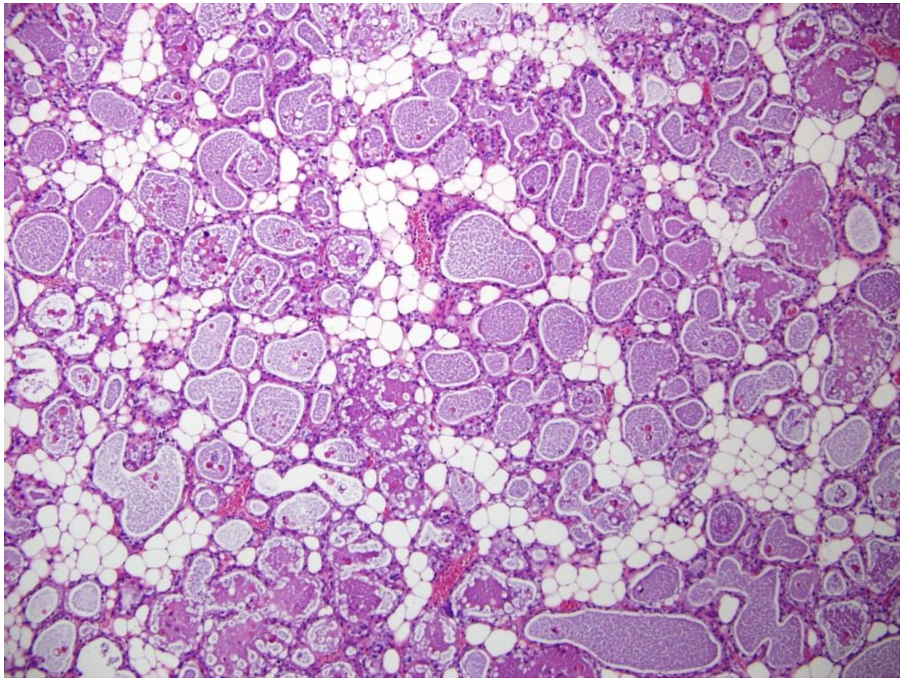

Stage 6: Dilated ducts; **Stage 7**: collapsed alveoli

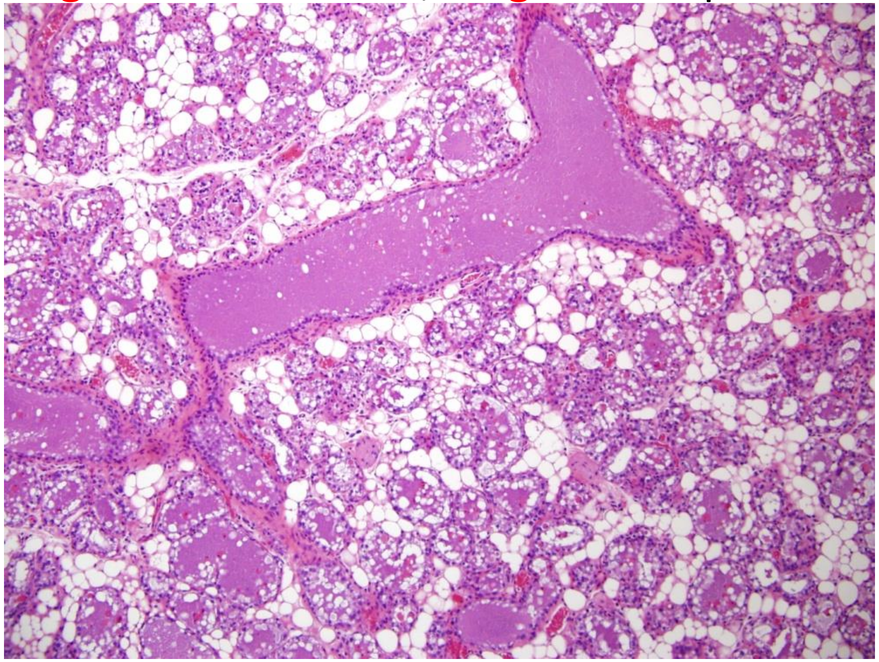

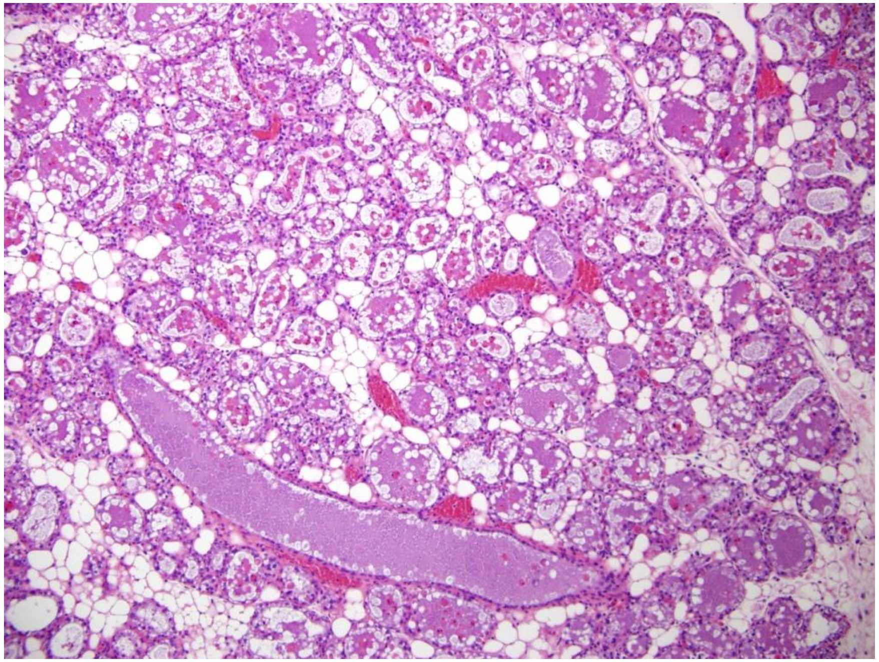

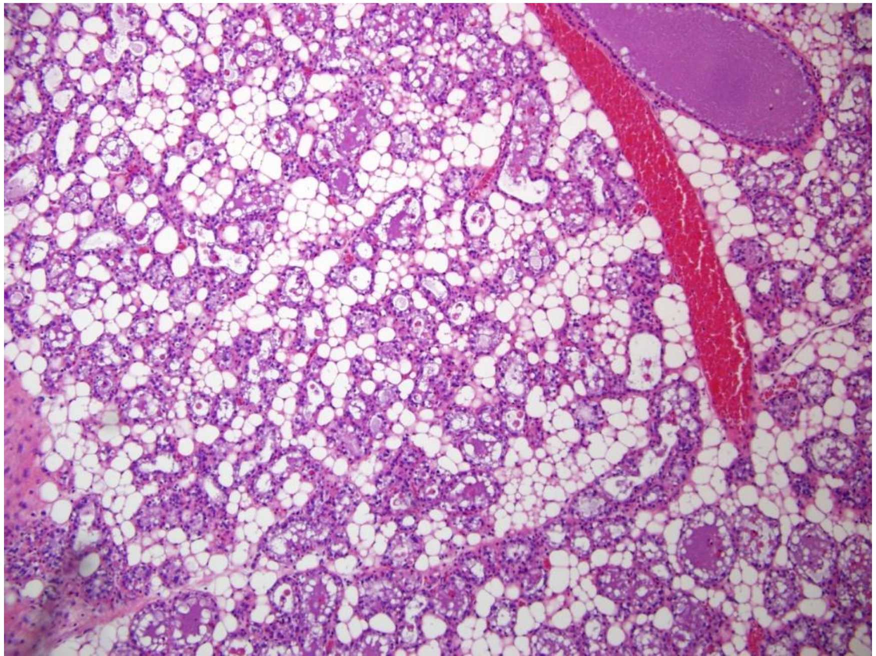

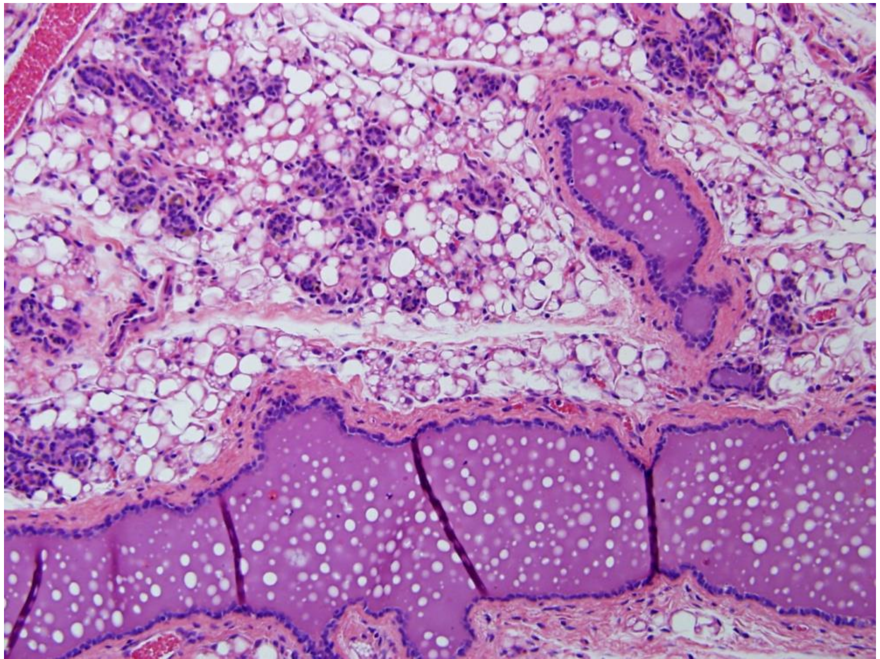

Stage 8: Fat pad restoring > replacing epithelium

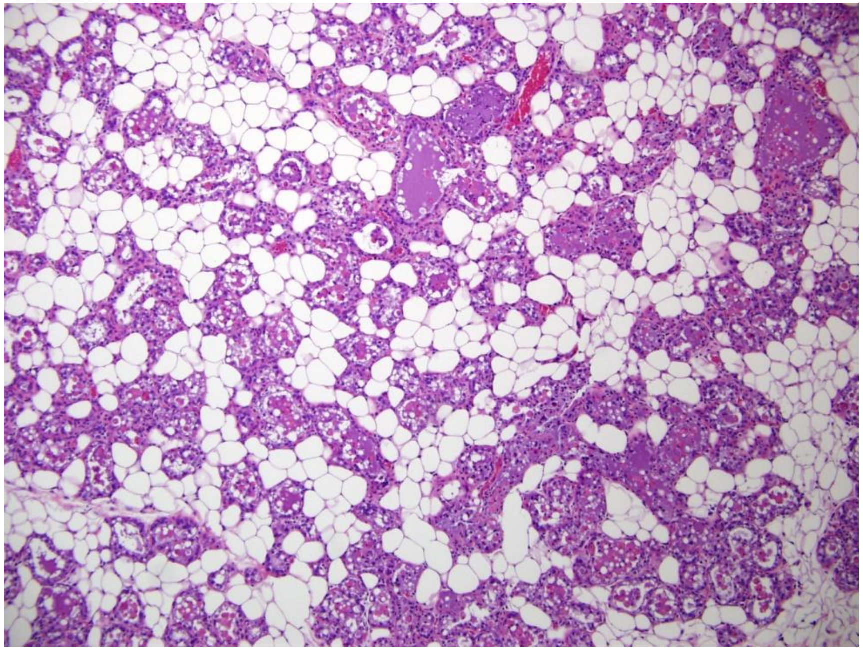

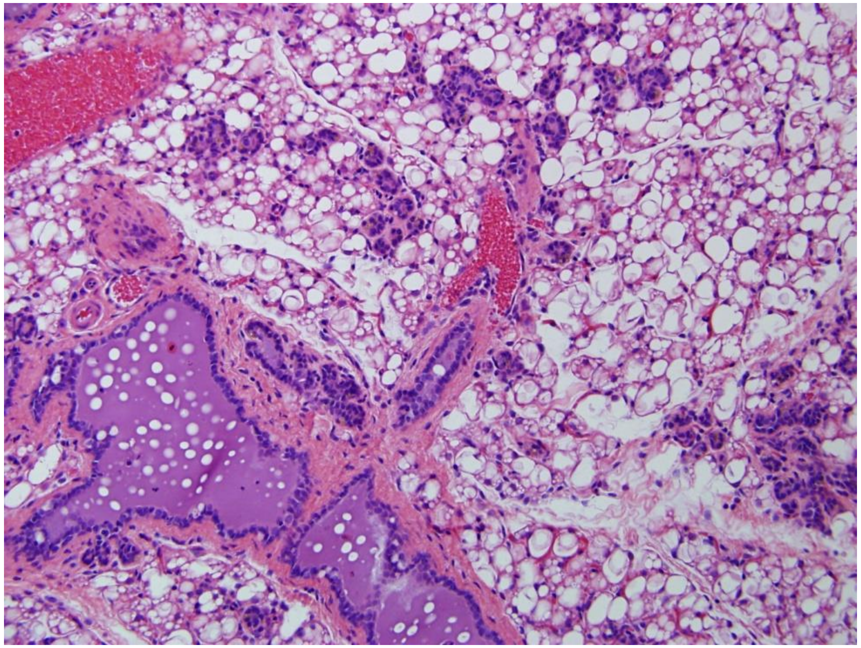

Supplement: Supplementary file 2 — Supplementary Figure S1 [file 41420_2018_105_MOESM2_ESM.pdf]
