# Supplementary material for: Autophagy and unfolded protein response (UPR) regulate mammary gland involution by restraining apoptosis-driven irreversible changes
Source: Cell Death Discov. 2018 Oct 15;4:40. doi: 10.1038/s41420-018-0105-y (PMC6186758; doi:10.1038/s41420-018-0105-y)

Supplementary Figure S2A

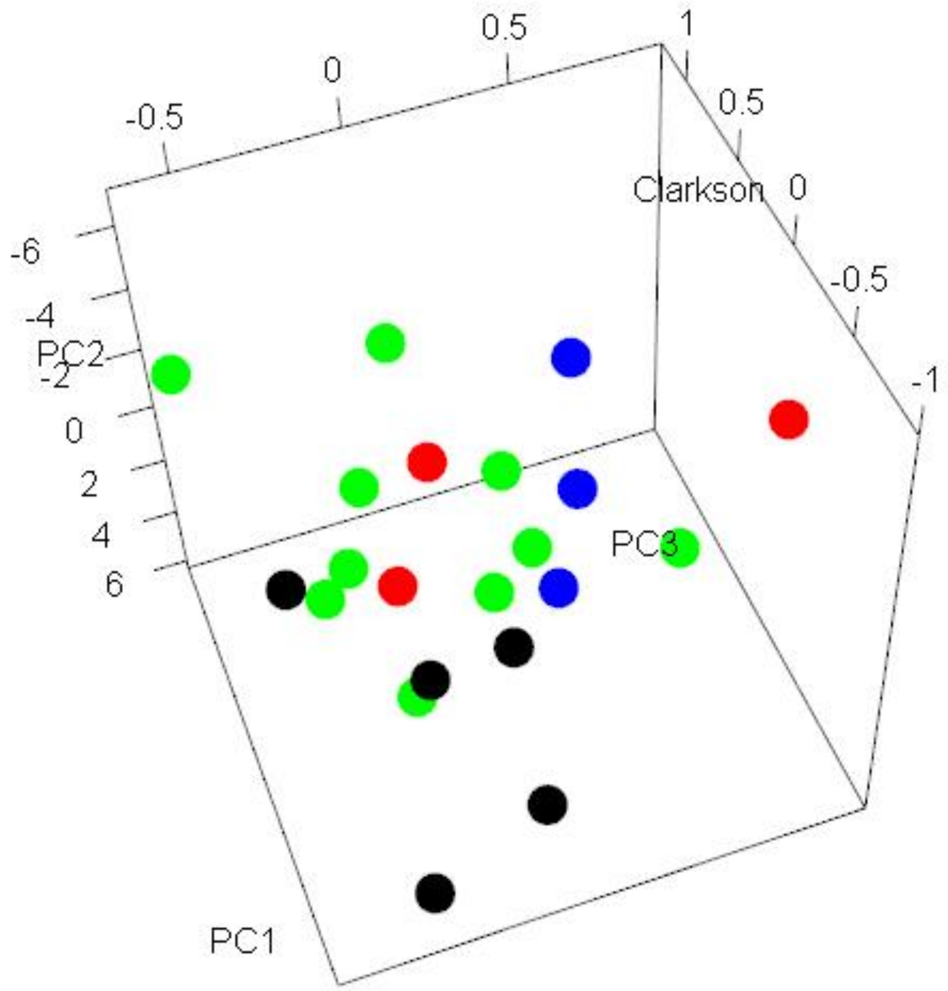

Supplementary Figure S2B

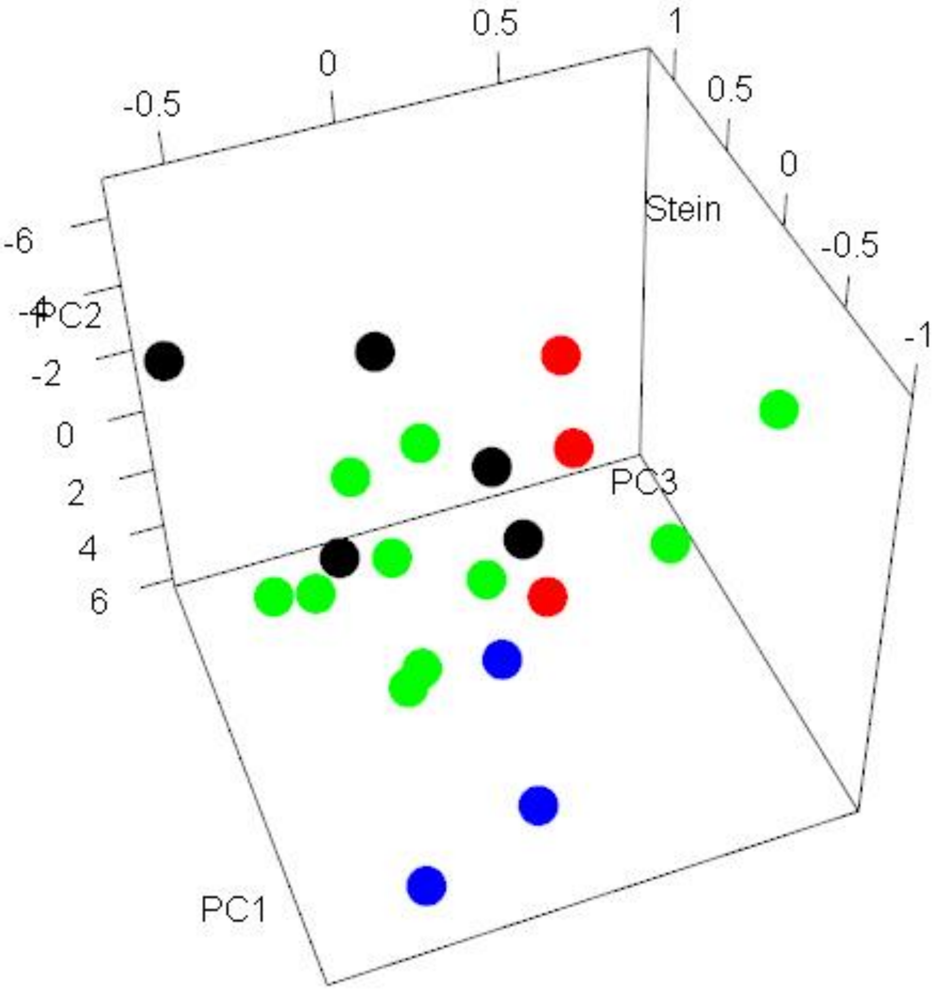

Supplementary Figure S3

Clarkson *et al.* 2004 data set

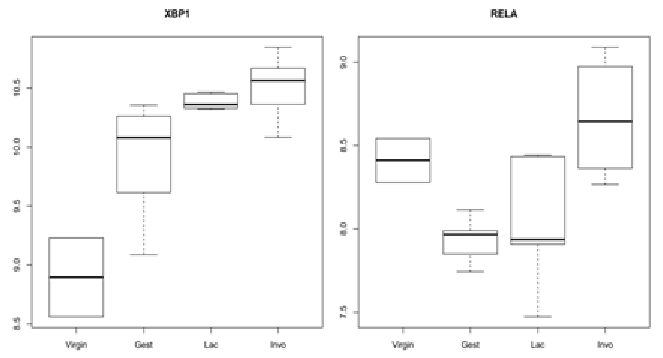

Stein *et al.* 2004 data set

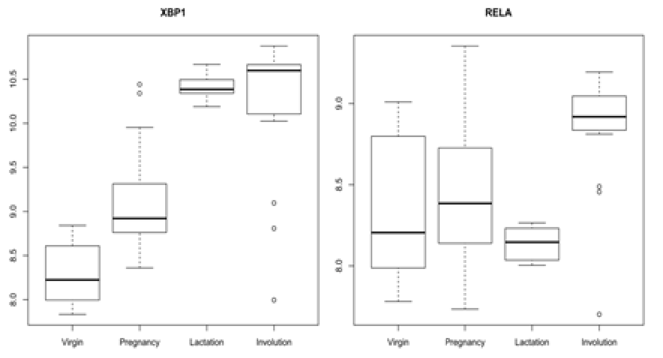

Supplement: Supplementary file 3 — Supplementary Figure S2-S3 [file 41420_2018_105_MOESM3_ESM.pdf]
