# Supplementary material for: Autophagy and unfolded protein response (UPR) regulate mammary gland involution by restraining apoptosis-driven irreversible changes
Source: Cell Death Discov. 2018 Oct 15;4:40. doi: 10.1038/s41420-018-0105-y (PMC6186758; doi:10.1038/s41420-018-0105-y)
Supplement: Supplementary file 4 — Supplementary Table S1 [file 41420_2018_105_MOESM4_ESM.docx]

| **Gene** | **Primer name** |
| --- | --- |
| Atg7 | **Atg7 IN13-F1** GCACTCTTACCTGAATGGCTGAG  **Atg7 IN14-R1** CAGTGGCCAGCCATTTCCAGC  **Atg7 IN13-R4** GCAAGCTCACTAGGCTGCAGAACC |
| LC3  amplifies the third intron of the LC3 genome as an internal control | **GFP1**  5′-TCCTGCTGGAGTTCGTGACCG-3′  **LC3**  5′-TTGCGAATTCTCAGC-CGTCTTCATCTCTCTCGC-3′  **mLC3ex3GT**  5′-TGAGC-GAGCTCATCAAGATAATCAGGT-3′  **mLC3ex4AG**  5′-GTTAGCATT-GAGCTGCAAGCGCCGTCT-3′) |

**Supplementary Table S1A.** Primer sequences for Atg7 and LC3 mouse genotyping.

| **Gene** | **Forward sequence** | **Reverse sequence** |
| --- | --- | --- |
| ACTβ | CTGTCCCTGTATGCCTCTG | ATGTCACGCACGATTTCC |
| AMBRA | GGGCAGTAATTGGAGATGGAC | GTACCAGGACATTCACAGAGG |
| ATF4 | ATGGCGTATTAGAGGCAGC | CTTTGTCCGTTACAGCAACAC |
| ATF6 | GAGGCTGGGTTCATAGACATG | GCTAGTGGTTTCTGTGTACTGG |
| ATG12 | ACCATCCAAGGACTCATTGAC | CCATCACTGCCAAAACACTC |
| ATG7 | TCTCCTACTCCAATCCCGTG | TGCTCATGTTGAACCCTCTG |
| BECLIN1 | ACACAGTCCAGAAAAGCTACC | GTACCGACTTGTTCCCTATGG |
| CHOP | TGTTGAAGATGAGCGGGTG | AGGTTCTGCTTTCAGGTGTG |
| CK18 | ACACCAACATCACAAGGCTG | TTCCACAGTCAATCCAGAGC |
| GRP78 | AGTTGATATTGGAGGTGGGC | CATTGAAGTAAGCTGGTACAGTAAC |
| GRP94 | AACCTCTGCTCAACTGGATG | TTGTGCCTTCATGATCCTCTC |
| HPRT | CCTCATGGACTGATTATGGACAG | TCAGCAAAGAACTTATAGCCCC |
| p62 | CCTATACCCACATCTCCCACC | TGTCGTAATTCTTGGTCTGTAGG |
| XBP1 | AAGAAAGCCCGGATGAGC | AGCGTGTTCTTAACTCCTGG |

**Supplementary Table 1B.** Quantitative real time reverse transcription PCR primer sequences.
