# Supplementary material for: Autophagy and unfolded protein response (UPR) regulate mammary gland involution by restraining apoptosis-driven irreversible changes
Source: Cell Death Discov. 2018 Oct 15;4:40. doi: 10.1038/s41420-018-0105-y (PMC6186758; doi:10.1038/s41420-018-0105-y)
Supplement: Supplementary file 5 — Supplementary Table S2 [file 41420_2018_105_MOESM5_ESM.docx]

|  |  |  |  |  |  |  |
| --- | --- | --- | --- | --- | --- | --- |
| **PCR Array** | **Human** | **total** | **expected** | **over/under** | **p-value** | **gene** |
| **Apoptosis** | 384 | 3 | 1.43 | + | 1.12E-03 | BCL2 SQSTM1 TSC1 |
| **Autophagy** | 384 | 10 | 0.22 | + | 3.43E-09 | AMBRA1 ATG7 BCL2L1 BECN1 MCL1 MTOR TSC1 ATG12 ATG5 BCL2L2 |
| **Unfolded Protein Response** | 384 | 8 | 0.08 | + | 1.76E-06 | ATF4 ATF6 DDIT3 EIF2A EIF2AK3 HSP90B1 HSPA5 XBP1 |

**Supplementary Table S2.**Selected genes representing the processes of apoptosis, autophagy and the unfolded protein response (UPR). We used our prior knowledge of signaling in breast cancer to create a list of genes that would likely represent adequately the processes of apoptosis, autophagy and the unfolded protein response. We then validated the selected gene set for this purpose using SABioscience PCR Array list and Fisher’s Exact test.
